# A Novel Vasoactive Proline-Rich Oligopeptide from the Skin Secretion of the Frog *Brachycephalus ephippium*

**DOI:** 10.1371/journal.pone.0145071

**Published:** 2015-12-14

**Authors:** Daniel Dias Rufino Arcanjo, Andreanne Gomes Vasconcelos, Simón Gabriel Comerma-Steffensen, Joilson Ramos Jesus, Luciano Paulino Silva, Osmindo Rodrigues Pires, Claudio Miguel Costa-Neto, Eduardo Brandt Oliveira, Ludovico Migliolo, Octávio Luiz Franco, Carolina Baraldi Araújo Restini, Michele Paulo, Lusiane Maria Bendhack, Marcelo Porto Bemquerer, Aldeidia Pereira Oliveira, Ulf Simonsen, José Roberto de Souza de Almeida Leite

**Affiliations:** 1 Núcleo de Pesquisa em Biodiversidade e Biotecnologia–BIOTEC, *Campus* Ministro Reis Velloso–CMRV, Universidade Federal do Piauí –UFPI, Parnaíba, PI, Brazil; 2 Laboratório de Farmacologia Cardiovascular–LFC, Núcleo de Pesquisas em Plantas Medicinais–NPPM, Universidade Federal do Piauí –UFPI, Teresina, PI, Brazil; 3 Pulmonary and Cardiovascular Pharmacology, Department of Biomedicine, Aarhus University, Aarhus, Denmark; 4 Laboratório de Espectrometria de Massa, EMBRAPA Recursos Genéticos e Biotecnologia, Brasília, DF, Brazil; 5 Laboratório de Toxinologia, Instituto de Ciências Biológicas–ICB, Universidade de Brasília–UnB, Brasília, DF, Brazil; 6 Departamento de Bioquímica e Imunologia, Faculdade de Medicina de Ribeirão Preto–FMRP, Universidade de São Paulo–USP, Ribeirão Preto, SP, Brazil; 7 Centro de Análises Proteômicas e Bioquímicas–CAPB, Universidade Católica de Brasília–UCB, Brasília, DF, Brazil; 8 Universidade de Ribeirão Preto–UNAERP, Curso de Medicina, Avenida Costábile Romano 2201, Ribeirão Preto, SP, Brazil; 9 Departamento de Física e Química, Faculdade de Ciências Farmacêuticas de Ribeirão Preto–FCFRP, Universidade de São Paulo–USP, Ribeirão Preto, SP, Brazil; Instituto Butantan, BRAZIL

## Abstract

Proline-rich oligopeptides (PROs) are a large family which comprises the bradykinin-potentiating peptides (BPPs). They inhibit the activity of the angiotensin I-converting enzyme (ACE) and have a typical pyroglutamyl (Pyr)/proline-rich structure at the N- and C-terminus, respectively. Furthermore, PROs decrease blood pressure in animals. In the present study, the isolation and biological characterization of a novel vasoactive BPP isolated from the skin secretion of the frog *Brachycephalus ephippium* is described. This new PRO, termed BPP-Brachy, has the primary structure WPPPKVSP and the amidated form termed BPP-BrachyNH_2_ inhibits efficiently ACE in rat serum. *In silico* molecular modeling and docking studies suggest that BPP-BrachyNH_2_ is capable of forming a hydrogen bond network as well as multiple van der Waals interactions with the rat ACE, which blocks the access of the substrate to the C-domain active site. Moreover, in rat thoracic aorta BPP-BrachyNH_2_ induces potent endothelium-dependent vasodilatation with similar magnitude as captopril. In DAF-FM DA-loaded aortic cross sections examined by confocal microscopy, BPP-BrachyNH_2_ was found to increase the release of nitric oxide (NO). Moreover, BPP-BrachyNH_2_ was devoid of toxicity in endothelial and smooth muscle cell cultures. In conclusion, the peptide BPP-BrachyNH_2_ has a novel sequence being the first BPP isolated from the skin secretion of the Brachycephalidae family. This opens for exploring amphibians as a source of new biomolecules. The BPP-BrachyNH_2_ is devoid of cytotoxicity and elicits endothelium-dependent vasodilatation mediated by NO. These findings open for the possibility of potential application of these peptides in the treatment of endothelial dysfunction and cardiovascular diseases.

## Introduction

**Fig 1:**
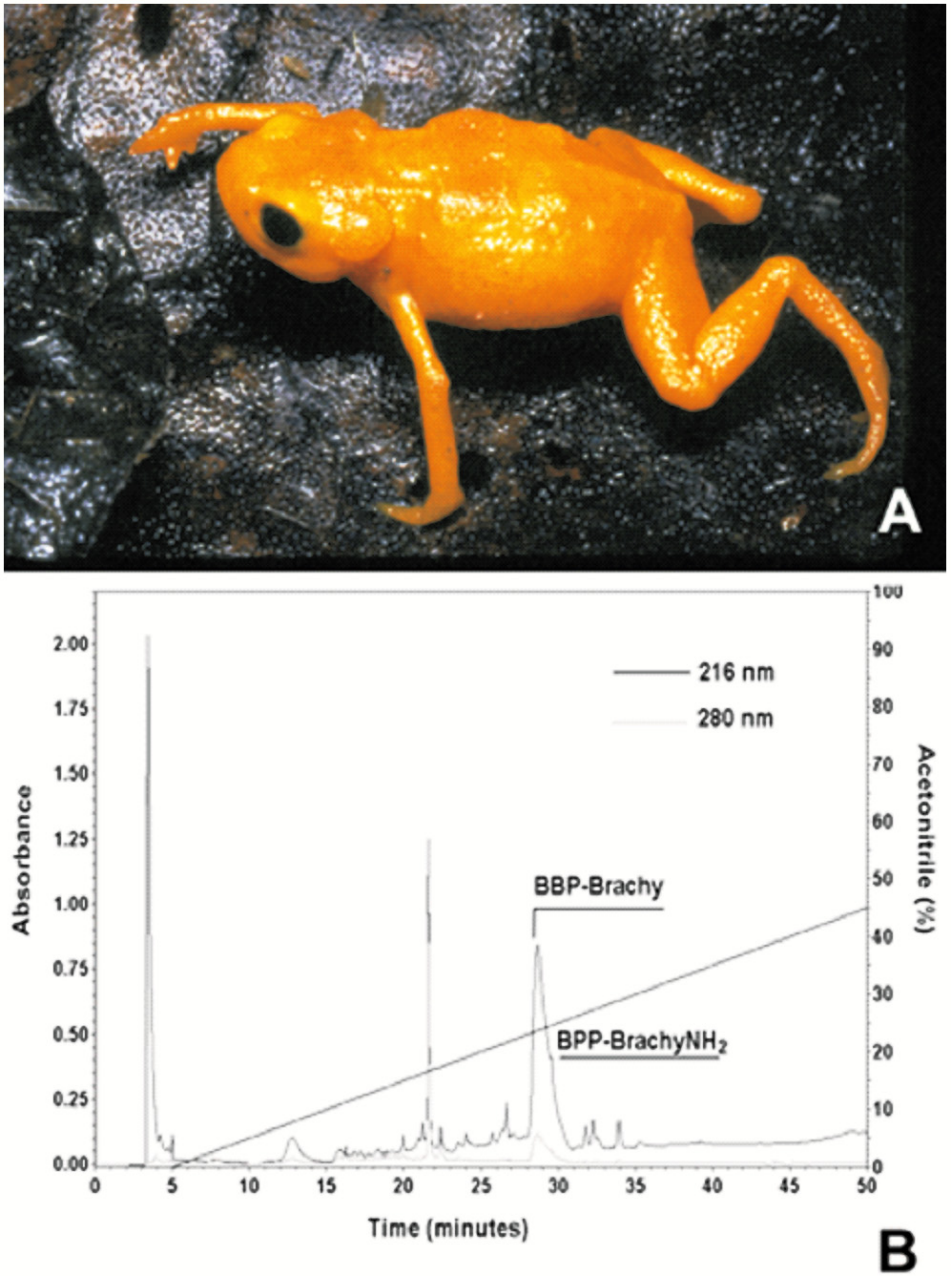
(A) Adult male of *Brachycephalus ephippium* (Spix, 1824) (Photo: Pombal Jr., J.P.). (B) Fractionation of peptides from crude skin secretion of *B*. *ephippium*. Sample containing 3.0 mg of lyophilized skin secretion was dissolved in 150 μL of 0.1% trifluoroacetic acid and loaded onto a Vydac C_18_ column; chromatography was carried out with a linear gradient of acetonitrile concentration on water from 0 to 100% in 0.1% trifluoroacetic acid, at flow rate of 1.0 mL/min for 60 min. Peptides in the effluent of the column were monitored by absorbance measurements at the indicated wavelengths.

The Brachycephalidae family is composed of 54 frog species, divided into two genera (*Brachycephalus* Fitzinger, 1826 and *Ischnocnema* Reinhardt and Lütken, 1862). Frogs of the Brachycephalidae family have been found in Southern and Central Brazil and adjacent Northern Argentina, and they are probably also present in the adjacent part of Paraguay (American Museum of Natural History. http://research.amnh.org/vz/herpetology/amphibia/Amphibia/Anura/Brachycephalidae - Retrieved on 07 Apr 2014). Among this family, *Brachycephalus ephippium* Spix, 1824 (Fig 1A) is a diurnal small frog (18 mm SVL [snout-vent length]) and presents an attractive yellow warning coloration [1]. Modeling of habitats of the *ephippium* cluster had been proposed for species closely related to *B*. *ephippium*, being the high elevation areas in the Serra do Mar, Brazil, one of the most suitable areas found [2]. Pires et al. have reported the identification of tetrodotoxin (TTX) and several analogues in the skin secretion of *B*. *ephippium* and of two other species from *Brachycephalus* genus [3,4]. Interestingly, a TTX-analogue called 11-oxotetrodotoxin, has been reported to be four to five-fold more toxic than TTX [5].

The bioactive peptides usually found in the skin secretion of several amphibians are often reported as an important defense strategy against predators. Among them, the proline-rich oligopeptides (PROs) are a large family, which include the bradykinin-potentiating peptides (BPPs), known as inhibitors of angiotensin I-converting enzyme (ACE, EC 3.4.15.1). Thus, in endothelial cells they inhibit the zinc metallopeptidase, which is able to convert inactive angiotensin I to the potent vasoconstrictor angiotensin II and degrades bradykinin (BK) into either inactive BK (1–7) or BK (1–5) [6,7].

The *Bj*-BPP-5a was the first BPP for which the amino acid sequence was described. *Bj*-BPP-5a is present in hydroalcoholic extracts of the snake venom *Bothrops jararaca* [8,9]. In contrast to oral administration, parenteral administration showed benefits of *Bj*-BPP-9a for the treatment of human hypertension [10]. Based on these observations, ACE inhibition was considered a pivotal target for treatment of hypertension, and together with a model of somatic ACE (sACE), a metallopeptidase with a zinc-binding carboxyl group at the catalytic center, led to design of captopril. Captopril was the first effective antihypertensive drug designed to bind and inhibit the active sites of ACE, and represented a breakthrough in the treatment of hypertension [11,12].

Interestingly, ACE inhibition is only one possible mechanism whereby animal toxins and BPPs may have a vasodilatory and antihypertensive effect. The synthetic peptides *Bj*-BPP-7a and -10c were suggested to have an ACE-independent antihypertensive effect [13], and the activation of the argininosuccinate syntethase enzyme (AsS) was proposed as a target for *Bj*-BPP-10c followed by increased L-arginine levels and formation of NO [14]. For *Bj*-BPP-5a, the increment of NO production depends on M_1_ muscarinic receptor and B_2_ bradykinin receptor activation [15], which supports the NO-dependent anti-hypertensive effect observed for this peptide [16]. For *Bj*-BPP-13a, the increase of NO production have been related to the M_3_ muscarinic receptors activation [17]. Thus, apart from ACE inhibition, animal toxins and BPPs have been reported to activate other enzymes and receptors which could be involved in the vasodilatory effect of BPPs, and lead to discovery of new targets with potential for therapeutic applications [18,19].

In the present study, we investigated the structure and vasodilatory properties of BPP-BrachyNH_2_ (WPPPKVSP), a novel proline-rich oligopeptide (PRO) isolated from the skin secretion of the pumpkin toadlet frog, *Brachycephalus ephippium*.

## Material and Methods

### Ethics Statement

The collection of frogs was authorized by the *Instituto Brasileiro do Meio Ambiente e dos Recursos Renováveis*, IBAMA-Brazil, under license number 02010.003041/05-87. After collection of the cutaneous secretion, the frogs were euthanized by 20% carbon dioxide, following the Resolution no. 1000/2012 from the Federal Council of Veterinary Medicine, Brazil. The death was confirmed by the absence of response to mechanical stimulus on the hind paws. The approval by an ethics committee for this purpose is not required in Brazil, but only the permission to collect the frogs, in concordance to the environmental legislation. Afterwards, the frogs were incorporated in the Brazilian Zoological collection.

Rats were handled and euthanized in accordance with Resolution no. 1000 (2012) of the Brazilian Federal Council of Veterinary Medicine, in order to minimize suffering. All procedures were approved by the local Ethics Committee for Animal Experimentation (*Universidade Federal do Piauí*, Brazil; permission number: 008/2012).

### Cutaneous secretion of frogs and purification of the peptides

Adult specimens of *B*. *ephippium* (n = 23) (Fig 1A) were collected in Mogi-Mirim, a region of the Brazilian Atlantic forest in São Paulo State. Cutaneous secretions from *B*. *ephippium* were obtained by a brief electric stimulation of the skin glands. The hydrophilic secretive fractions were injected into an analytical Vydac reversed-phase column (150 mm × 4.6 mm, C_18_, 5 μm, 218TP104) in a High Performance Liquid Chromatography (HPLC) analytical system (Shimadzu Co., Kyoto, Japan). The purification was performed at room temperature and under 0 to 100% gradient of acetonitrile (ACN) in 0.1% trifluoroacetic acid (TFA) for 60 min with UV detection at 216 and 280 nm [20].

### Mass spectrometry analysis and *De novo* sequencing

The molecular masses and homogeneity of BPPs (BPP-Brachy and BPP-Brachy-NH_2_) were determined by UltraFlex III MALDI-TOF/TOF (Bruker Daltonics, Billerica, MA, USA) in an α-cyano-4-hydroxycinnamic acid matrix, similar to Machado and colleagues, but with modifications [21]. The mass spectrometer was operated in reflector positive mode for MS or LIFT™ and positive mode for MS/MS experiments by FlexControl™ software. Instrument calibration was performed externally with [M+H]^+^ ions of angiotensin I, angiotensin II, substance P, bombesin, and adrenocorticotropic hormones (fragments 1–17 and 18–39). Accumulated data from 200 consecutive laser shots were acquired for each spectrum. Samples were analyzed by both MALDI-TOF and LIFT™ MALDI-TOF/TOF MS/MS from the same target. The ion spectra were manually interpreted by *De novo* sequencing. For determination of isomeric and isobaric residues, the high-energy fragmentation was used [22]. A Search for peptide sequence alignments and similarities were performed by using the FASTA 3 program on the ExPASy molecular server (http://www.expasy.ch/).

### Peptide synthesis

The synthesis of the octapeptide BPP-BrachyNH_2_ was carried out manually, with a standard Fmoc (N-(9-fluorenyl)methoxycarbonyl) chemistry [23] starting from a Rink-amide-MBHA resin (0.59 mmol.g^-1^, Peptides International, Louisville, KY, USA). Fmoc-protected amino acids (Peptides International, Louisville, KY, USA) were used in four-fold molar excess relative to the nominal scale of synthesis (1.2 mmol). Couplings were performed with 1,3-diisopropylcarbodiimide/ethyl 2-cyano-2-(hydroxyimino) acetate (DIC/Oxyma) in N,N-dimethylformamide (DMF) for 2-3h. Side chain protected groups were tert-butyl for Ser, and Boc for Lys and Trp. Deprotected groups were conducted by 4-methylpiperidine/DMF (1:4, v:v) for 20–30 min. Removal of side chain protection and cleavage of the peptide from the resin were performed by the use of 10.0 mL TFA:water:tioanisol:ethanodithiol:triisopropylsilane (86:5.0:5.0:2.5:1.0, v:v:v:v:v) with addition of 1 g phenol for 90 min at room temperature under shaking. After solvent evaporation under nitrogen, the peptide was precipitated by addition of cold diisopropyl ether, collected by filtration and washed four times with cold diisopropyl ether. Extraction was performed with 200 mL H_2_O:ACN (1:1, v:v) and crude peptide was lyophilized. Purification was performed using a Shimadzu HPLC system fitted with a Vydac C_18_ column (150 × 4.6 mm) developed with a linear ACN gradient (12–35%; 25 min) in 0.05% TFA. Purity and identity were verified by the use of MALDI-TOF MS and MS/MS. Stock peptide solutions were prepared in water and their concentrations were determined according to tryptophan molar absorptivity (5550 M^-1^.cm^-1^) at 280 nm.

### ACE inhibition assay

The inhibitory effects of BPP-BrachyNH_2_ and captopril on the ACE-catalyzed hydrolysis of hippuryl-His-Leu-OH were estimated in presence of increasing concentrations of inhibitors (BPP-BrachyNH_2_ from 0.05 to 50 μM; captopril from 3 × 10^−5^ to 2 μM). Fresh Wistar rat serum was used as source of ACE in these reactions, and the product H-His-Leu-OH was measured fluorimetrically following derivatization with *o*-phtaldialdehyde, as described [24,25]. Reactions were carried out, in duplicate, at 37°C for 30 min in 200 μL of 20 mM Tris-HCl buffer, pH 8.1, 0.3 M NaCl, 1.0 mM H-hippuryl-His-Leu-OH, 20 μL of enzyme-containing serum and different inhibitor concentrations as described above. The IC_50_ values, corresponding to the concentration of the inhibitor that results in 50% of maximal activity, were derived from fractional activity data plotted as a function of each inhibitor.

### Molecular modeling and *in silico* docking studies

The three-dimensional models for ACE from *Rattus norvegicus* (GenBank: AAG35596.1) and BPP-Brachy were constructed based on the structures of *Homo sapiens* ACE (UniProtKB ID: P12821; PDB code: 2YDM) and CDK2 (UniProtKB ID: P24941; PDB code: 3QTS), respectively. The 2YDM presents the structure of angiotensin I-converting enzyme from *Homo sapiens* ACE, resolved by X-ray diffraction with a resolution of 2.44 Å [26]. This structure was used as a template for the construction of a model for *Rattus norvegicus* ACE. The 3QTS presents the structure of a cyclin-dependent kinase 2 from *Homo sapiens* [27]. This structure was used as a template for the construction of BPP-Brachy.

Two hundred theoretical tridimensional peptide structures were constructed using Modeller v.9.12 for each peptide. The ACE and BPP-Brachy final models, i.e., geometry, stereochemistry, and energy distributions in the models, were evaluated using PROSA II to analyze packing and solvent exposure characteristics and PROCHECK for additional analysis of stereochemical quality. In addition, RMSD was considered by overlap of Cα traces and backbones onto the template structure by the use of the program 3DSS. The protein and peptide structures were visualized and analyzed on Delano Scientific’s PyMol (http://pymol.sourceforge.net/).

All docking calculations were performed using AUTODOCK 4.2 program. Docking simulation of BPP-Brachy was performed toward ACE C- and N-domain. All polar hydrogen atoms were added using the AutoDockTool. Grid maps were calculated with 30 × 30 × 30 Å for both ACE domains and 1.0 Å spacing centered in the active sites of the enzyme characterized as C- and N-domain, allowing interaction with all side chains exposed [28]. A Lamarckian genetic algorithm was used as the search method to find the best peptide–enzyme complex. Ten docking runs were done generating ninety models, where the maximum freedom to side chains was unlocked to the peptide. The generated structures were ranked in two steps: firstly a cluster with the best models with lowest energy, and secondly with a root-mean-square deviation (RMSD), for all atoms docked with the ACE C- or N-domain, showing tolerance of 4 Å, as recommended for blind docking [29]. The program PyMol (http://pymol.sourceforge.net/) was used to characterize peptide-enzyme interactions.

### Effect on vascular reactivity of rat thoracic aorta

Male Wistar rats (250 ± 30 g) were maintained under controlled temperature (22 ± 1 ≡C), 12/12 h light/dark cycle, and had free access to water and food (Purina-Nestlé, São Paulo, SP, Brazil). Rat thoracic aortic rings were prepared according to Silva-Filho and colleagues [30]. Briefly, aortic rings (3–4 mm) were maintained in Krebs solution (in mM: NaCl, 118.0; KCl, 4.6; CaCl_2_.2H_2_O, 2.5; MgSO_4_.7H_2_O, 5.7; NaHCO_3_, 25.0; KH_2_PO_4_.H_2_O, 1.1; and D-glucose, 11.0) under isometric tension of 1.0 g, 37°C and bubbled with 5% CO_2_ in 95% O_2_. The endothelium was considered as functionally intact if acetylcholine (10^−6^ M) induced relaxations larger than 70% in phenylephrine (PE 3 × 10^−7^ M)-contracted preparations. After washout, the preparations were contracted with PE (PE 3 × 10^−7^ M) and concentration-response curves for BPP-BrachyNH_2_ and captopril (10^−9^–3 × 10^−5^ M) were constructed. Time control contractions induced by PE, but without adding drug were obtained in parallel with other vascular segments.

### NO measurement on aortic rings by confocal microscopy

The fluorescence measurements of nitric oxide (NO) were conducted in freshly isolated aortic rings according to the method by Capellini and colleagues [31]. After the adherence to a slide, the aortic rings were loaded with 4-amino-5-methylamino-2',7'-difluorofluorescein diacetate (DAF-FM DA) and maintained under 5% CO_2_ (20 min, 37°C). Afterwards, the preparations were excited at 488 nm and fluorescence emitted was measured at 515 nm. The stock solution of the DAF-FM DA was prepared at 5 mM in dimethyl sulfoxide (DMSO), and the work solution (5 μM) was prepared by diluting the stock solution into Hanks solution (in mM: CaCl_2_, 1.6; MgSO_4_, 1.0; NaCl, 145.0; KCl, 5.0; NaH_2_PO_4_, 0.5; dextrose, 10.0; and HEPES, 10.0; pH 7.4). Fluorescence intensity was measured by use of a confocal laser microscope (Leica TSC SP5, Leica Microsystems, Wetzlar, Germany).

The preparations were stimulated during 100 seconds with Hanks solution, and thereafter they were stimulated with BPP-BrachyNH_2_ (10^−7^ or 10^−5^ M). The regions of interest (ROI) were selected and the intracellular fluorescence intensity was measured before and after the addition of BPP-BrachyNH_2_. The average fluorescence intensity was calculated for each animal (n). From these data, the initial fluorescence value at t = 100s was taken as the basal fluorescence (F_0_ = 100%), and the final fluorescence intensity value (F) was obtained, before and at t = 200s after BPP-BrachyNH_2_ addition.

### Cytotoxicity study

#### Cell culture

Human umbilical vascular endothelial cells (HUVECs) (ATCC, Manassas, VA, USA) were grown in Dulbecco's Modified Eagle Medium (DMEM) containing 40% of fetal bovine serum (FBS). Rat aortic vascular smooth muscle cells (VSMCs) were obtained from isolated rat aorta rings, that were longitudinally opened and its intimae layer exposed, in a six-well culture plate with culture medium under CO_2_ for 30 min. Then, 10 μL of culture medium containing streptomycin 10000 UI and 0.1% fungizone were added, and the tissue was submitted to cell migration and adhesion for two days.

#### MTT Assay

The cell viability after exposure to BPP-BrachyNH_2_ was measured, by 2-(3,5-diphenyltetrazol-2-ium-2-yl)-4,5-dimethyl-1,3-thiazole bromide (MTT) assay on HUVECs and VSMCs as described by Paulo and colleagues [32], with some modifications. Cells were plated into 96-well plates at 2 × 10^4^ cells/well, then a volume of 200 μL of culture medium was added, and the cell culture was incubated at 37°C under 5% CO_2_ for 24 hours. The culture medium was exchanged, and the cells were incubated with BPP-BrachyNH_2_ (10^−12^, 3 × 10^−9^, 10^−7^ and 10^−5^ M) for 24 hours. Afterwards, the medium was removed and replaced by phosphate buffered saline (PBS), 20 μL of MTT solution (5 mg mL^-1^) was added to 180 μL DMEM for each well. After incubation for 4 hours, the medium was replaced with 200 μL of DMSO, in order to dissolve the formazan crystals. The optical density was measured at 570 nm. The absorbances obtained for untreated cells and 1.0% Triton X-100-treated cells were taken as controls of cell viability and cytotoxicity, respectively.

### Statistical analyses

The IC_50_ values for the ACE activity and the pD_2_ values for the vasodilator curves were obtained by non-linear regression. All values were expressed as means ± SEM, and significances for compared values were analyzed by Student’s “t” test or One-way ANOVA followed by Bonferroni’s post-test. All procedures were performed using Graph Pad Prism 5.02™ (Graph Pad Software, Inc., San Diego, CA, USA).

## Results

### Identification of BPP-Brachy and BPP-BrachyNH_2_

**Fig 2:**
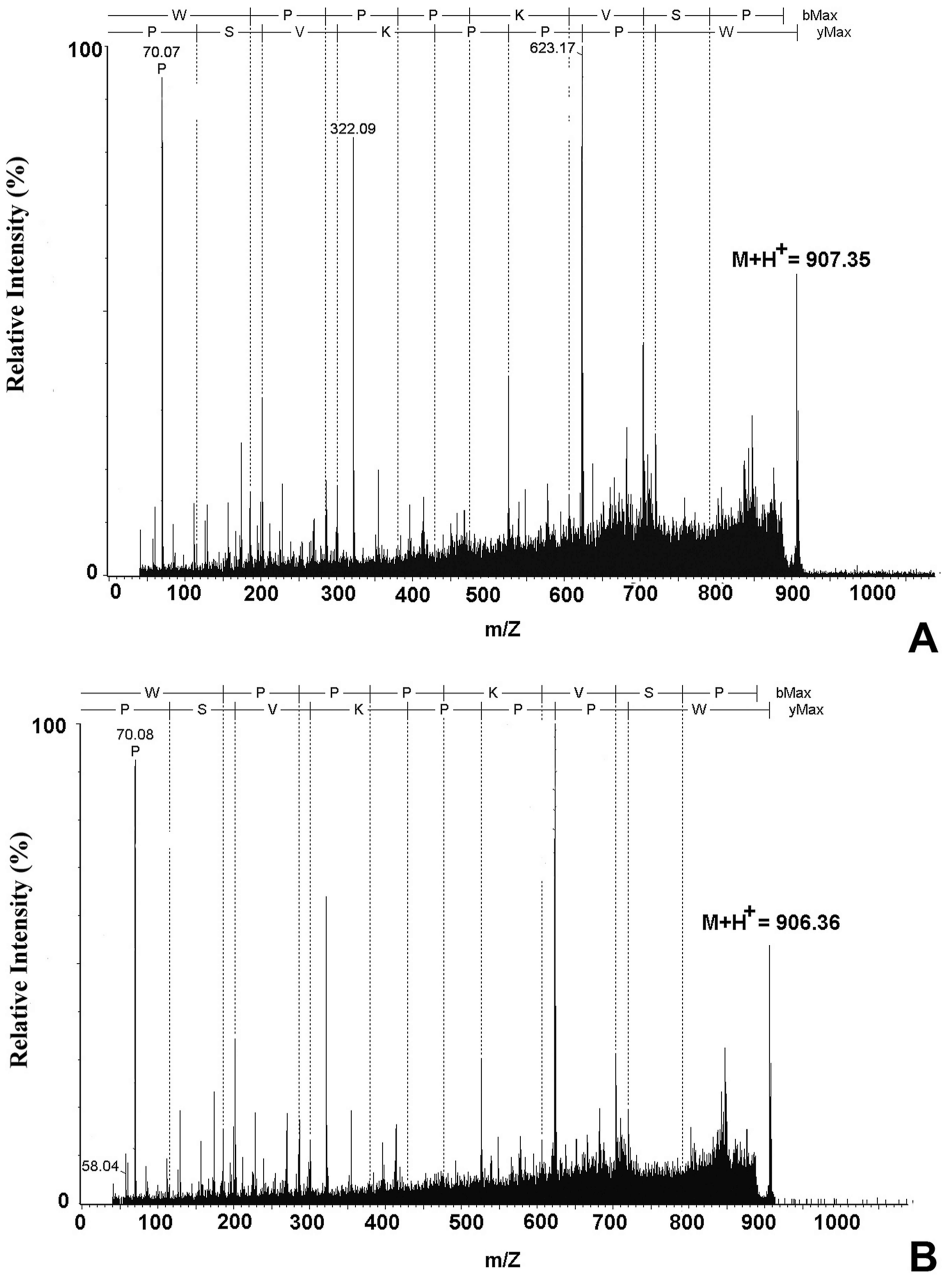
Sequencing of the proline-rich peptides (PROs) from the skin secretion of *B*. *ephippium*. (A) Mass spectra of BPP-Brachy, [M+H]^+^ = 907.37 and (B) BPP-BrachyNH_2_, [M+H]^+^ = 906.36 acquired in an UltraFlex III MALDI-TOF/TOF operating under LIFT™ mode for MS/MS experiments. The observed fragments allowed complete assignment of the major y and b ion series. The peptide sequence using one-letter code following the y and b series orientation is shown on the top part of the spectra.

The lyophilized crude skin secretion from *B*. *ephippium* (Fig 1A) was fractionated by analytical RP-HPLC, as shown in Fig 1B. Several peaks along the profile were detected and the arrows indicate the novel BPP and the amidated form thereof. This was further elaborated and confirmed by MS and MS/MS experiments. The primary structures WPPPKVSP (BPP-Brachy) and the amidated form thereof (BPP-BrachyNH_2_) were obtained after *De novo* sequencing by interpreting the MS/MS spectra (Fig 2). The compound with retention time of 22.5 minutes corresponds to the carotenoid β-carotene, which may be related to aposematic coloration of this species. The body color of lower vertebrates is determined by the types of chromatophores in the skin, and melanophores appear first in the dorsal integument of the larvae stage during the initial development [33].

### *In vitro* Evaluation of inhibitory ACE activity

**Fig 3:**
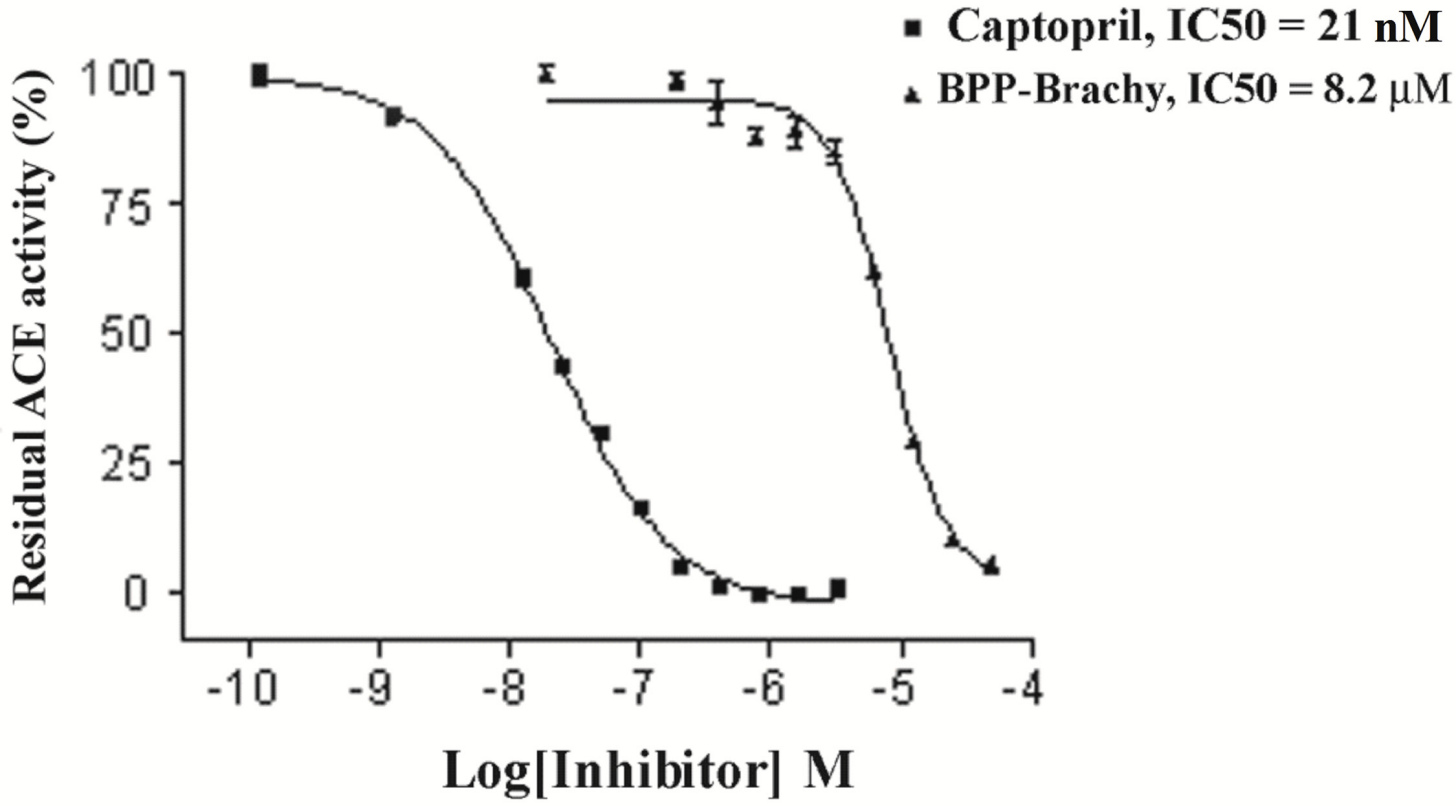
The inhibitory effect of BPP-BrachyNH_2_ and captopril on rat serum ACE activity. Residual enzymatic activities are plotted against the corresponding inhibitor concentrations. IC_50_ values were calculated from nonlinear regression analysis of obtained data using GraphPad 5.0 software (GraphPad Prism, San Diego, CA).

In order to test if BPP-BrachyNH_2_ was active in a mammalian system, the effect of the peptide was assessed on ACE activity in rat serum (Fig 3). Although BPP-BrachyNH_2_ (IC_50_ = 8.2 μM) had lower efficacy than captopril (IC_50_ = 21 nM) as ACE inhibitor, the fact that it functions as an ACE inhibitor and its distinct primary structure as compared with classical BPPs, prompted structure/function research with synthetic analogs.

### *In silico* Evaluation of inhibitory ACE activity

**Fig 4:**
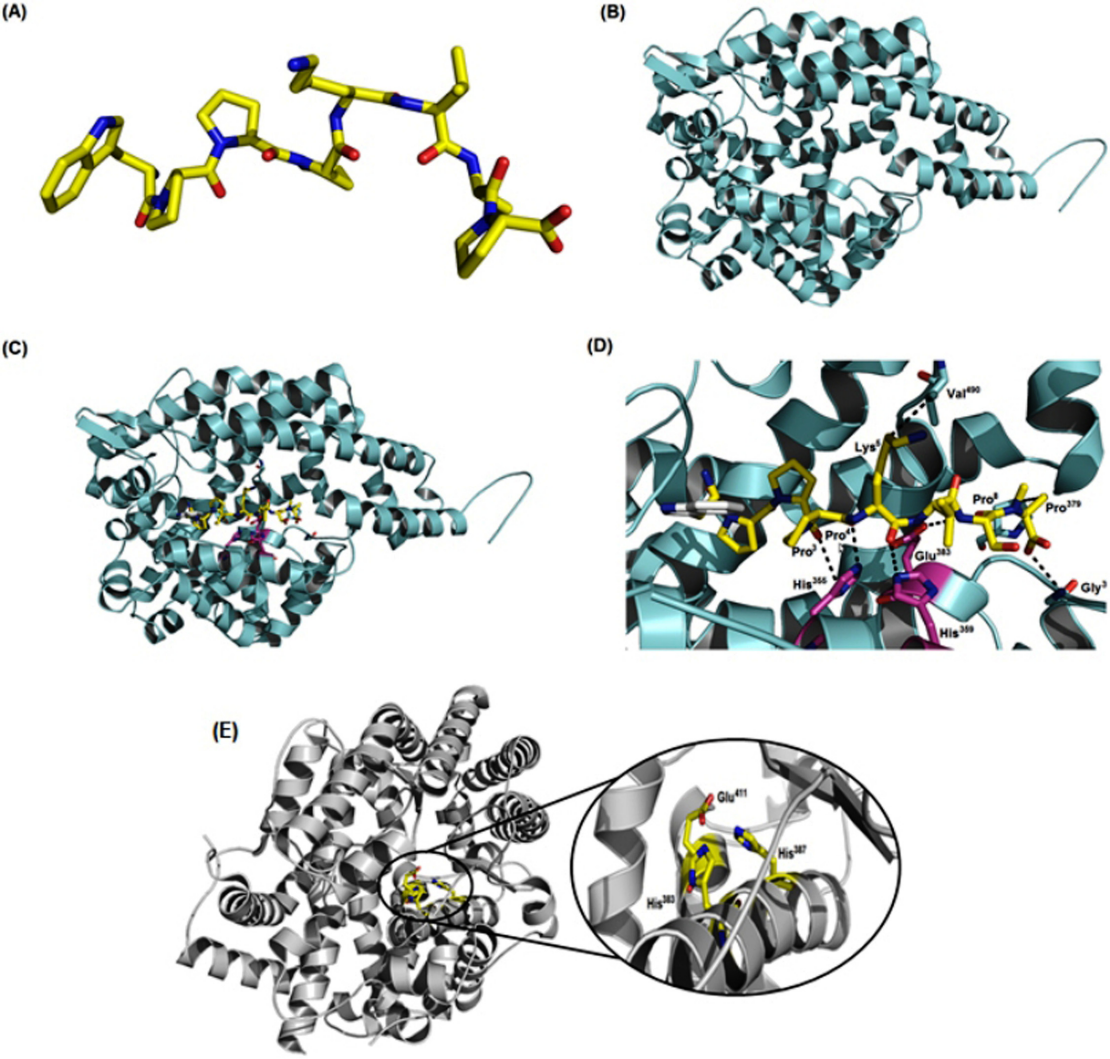
Molecular modeling of BPP-BrachyNH_2_ and human ACE and *in silico* docking studies. (A) Theoretical model of BPP-BrachyNH_2_ showing the structure with lower energy system; (B) theoretical model of ACE showing the structure with lower energy system; (C) Docking between BPP-BrachyNH_2_/ACE; (D) binary complex relationship with zoom in and detailed interactions; and (E) interactions between the substrate and the catalytic triad His^383^, His^387^ and Glu^411^, indicating a probable block of the catalytic activity.

The three-dimensional models of BPP-Brachy and ACE showed 50 and 83% of identity with 3qts and 2ydm, respectively. The cyclin-dependent kinase 2 from *Homo sapiens* was used as a template for the BPP-Brachy theoretical model, due to its high sequence identity (Fig 4A). The model for ACE was generated from the angiotensin I-converting enzyme of *Homo sapiens*, a structure that was resolved by X-ray diffraction with a resolution of 2.44 Å (Fig 4B). Validation of the 3D models by Ramachandran plot showed that the models presented a 100% of amino acid residues in allowed regions. The root main square deviation (RMSD) values for both BPP-Brachy and ACE models were 2.3 and 0.51 Å, respectively.

Molecular dockings were performed between BPP-Brachy and the two catalytic sites located in a region denominated N- and C-domain (Fig 4C). The interactions observed for BPP-Brachy and the C-domain presented energy of -9.1 kcal.mol^-1^. The complex BPP-Brachy/C-domain was stabilized among carbonyls of Pro^3^, Pro^4^ and Lys^5^ and hydrogens of nitrogen atoms from the imidazole rings of His^355^ and His^359^, forming a net of hydrogen bonds with distances of 3.7, 2.8, and 3.2 Å, respectively. BPP-Brachy also formed two possible hydrophobic contacts between the carbons of Pro^8^ and Lys^5^ (CE and CD) and the carbons (CG and CG) of the residues Pro^379^ and Val^490^, with distances of 3.5 and 3.0 Å, respectively. Hydrogen bonds were also observed between the hydrogen of nitrogen of Ser^7^ from BPP-Brachy and oxygen backbone atom of Glu^383^ from ACE, with a distance of 3.2 Å, as well as between the hydrogen of oxygen of Pro^8^ from BPP-Brachy and the nitrogen backbone atom of Gly^376^ from ACE, with a distance of 3.1 Å (Fig 4D). The histidine residues seem to be responsible for interactions between the substrate and the catalytic triad His^383^, His^387^ and Glu^411^, indicating a probable inhibition of the catalytic activity in a canonical fashion style, disallowing the generation of hypertensive peptide angiotensin II, by inhibiting the activity of angiotensin I-converting enzyme (ACE) (Fig 4E) [28,34]. In addition, BPP-Brachy was also docked to a second catalytic site in order to better understand the mechanism of inhibition. Nevertheless, the energy observed for the interaction between BBP-Brachy and N-terminal domain was -6.1 kcal.mol^-1^ (data not shown), lower than the interaction energy observed at the C-domain. Another critical point was the absence of interaction with important amino acid residues involved in catalytic activity of N-domain of human ACE (Tyr^369^ and Arg^389^). These *in silico* evidence reinforce that the ACE-inhibiting property of BPP-Brachy can be ascribed to a C-domain interaction rather than the N-domain, demonstrating lower affinity in the catalytic region.

### Vasodilator effect in rat aortic rings

**Fig 5:**
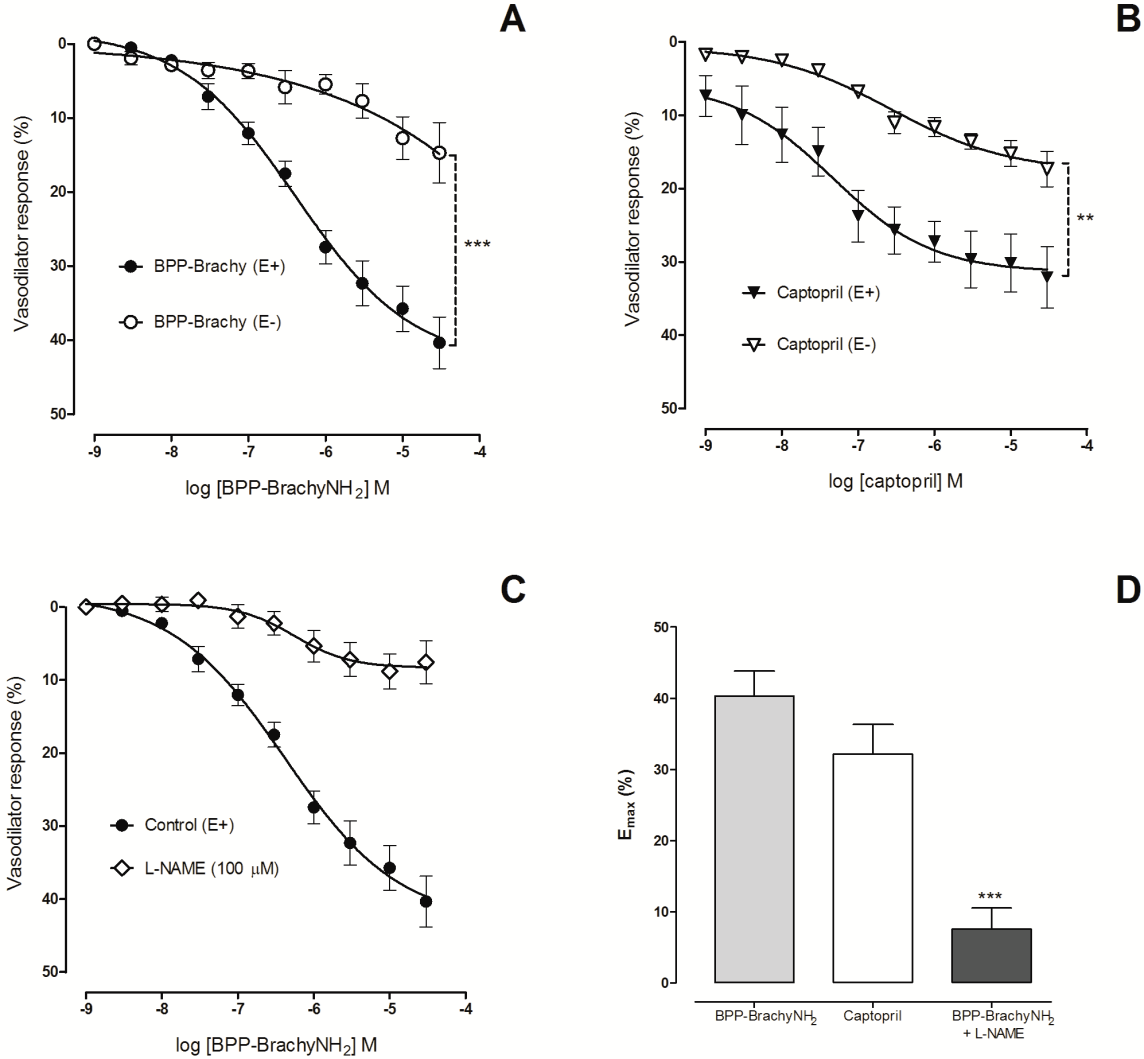
Vasodilator effect of BPP-BrachyNH_2_ and captopril (10^−9^–3 × 10^−5^ M) on rat thoracic aorta. Aortic rings were pre-contracted with phenylephrine (3 × 10^−7^ M) and then cumulatively incubated with BPP-BrachyNH_2_ (A) or captopril (B). Effect of L-NAME (100 μM) on BPP-BrachyNH_2_-induced vasodilator effect (C). Respective comparisons among E_max_ (D) values were plotted. The results were expressed as means ± SEM (n = 6). Non-paired Student’s t test. ^**^ *p* < 0.01 and ^***^ *p* < 0.001 versus endothelium-intact (E+) preparations.

BPP-BrachyNH_2_ induced pronounced concentration-dependent relaxation in preparations with endothelium (E_max_ = 40.3 ± 3.5%), while the effect was minimal in preparations without endothelium (E_max_ = 14.7 ± 4.1%) (Fig 5A). The reference drug captopril induced also endothelium-dependent vasodilatation with the same maximal relaxation (E_max_ = 32.1 ± 4.2%) as BPP-BrachyNH_2_ (Fig 5B–5D). The aortic preparations were washed and stabilized during 30 min, and then a new PE-induced vasoconstriction was evoked. PE induced vasoconstriction with the same potency, indicating that the effect of BPP-BrachyNH_2_ in aortic cross sections is reversible and non-lethal to vascular cells (data not shown).

The involvement of NO in BPP-BrachyNH_2_-induced relaxation was evaluated by inhibition of endothelial NO synthase with L-NAME [35]. In the presence of L-NAME, BPP-BrachyNH_2_ relaxation was abolished (Fig 5C), and the E_max_ value was decreased 5.3-fold (E_max_ = 7.6 ± 2.9%) (Fig 5D), suggesting the involvement of NO in BPP-BrachyNH_2_-induced relaxation in rat thoracic aorta.

### NO measurements by laser confocal microscopy

**Fig 6:**
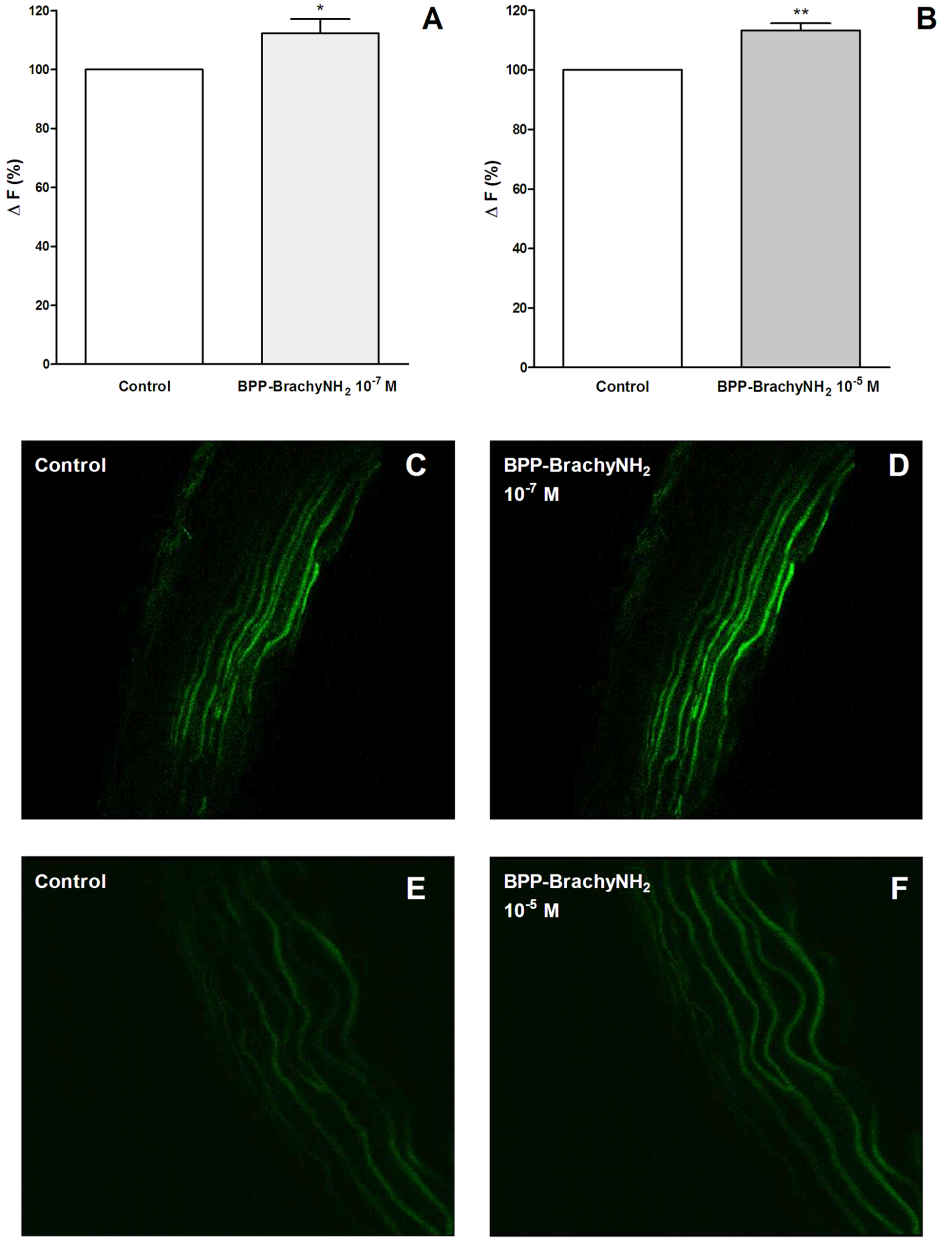
NO measurement after BPP-BrachyNH_2_ addition on endothelium-intact rat aorta cross sections assessed by using a confocal scanning laser microscope. (A) Fluorescence emission intensity for BPP-BrachyNH_2_ 10^−7^ M and (B) BPP-BrachyNH_2_ 10^−5^ M. Representative confocal photomicrograph of aortic cross sections loaded with DAF-FM DA (5 μM) before and after addition of BPP-BrachyNH_2_ 10^−7^ M (C-D) or 10^−5^ M (E-F). Results are reported as mean ± SEM (n = 4). Paired Student’s t test, ^*^ *p* < 0.05 and ^**^ *p* < 0.01 versus control.

To directly measure whether BPP-BrachyNH_2_ increase NO release from the endothelium, aortic segments were loaded with a NO-sensitive probe, DAF-FM DA. Incubation with BPP-BrachyNH_2_ increased fluorescence with, respectively, 12.3 ± 4.8% and 13.2 ± 2.4% in response to 10^−7^ and 10^−5^ M of BPP-BrachyNH_2_ (Fig 6). These results support that BPP-BrachyNH_2_ increases NO and that NO mediates the endothelium-dependent relaxations of the peptide.

### Cytotoxic evaluation in vascular cells

**Fig 7:**
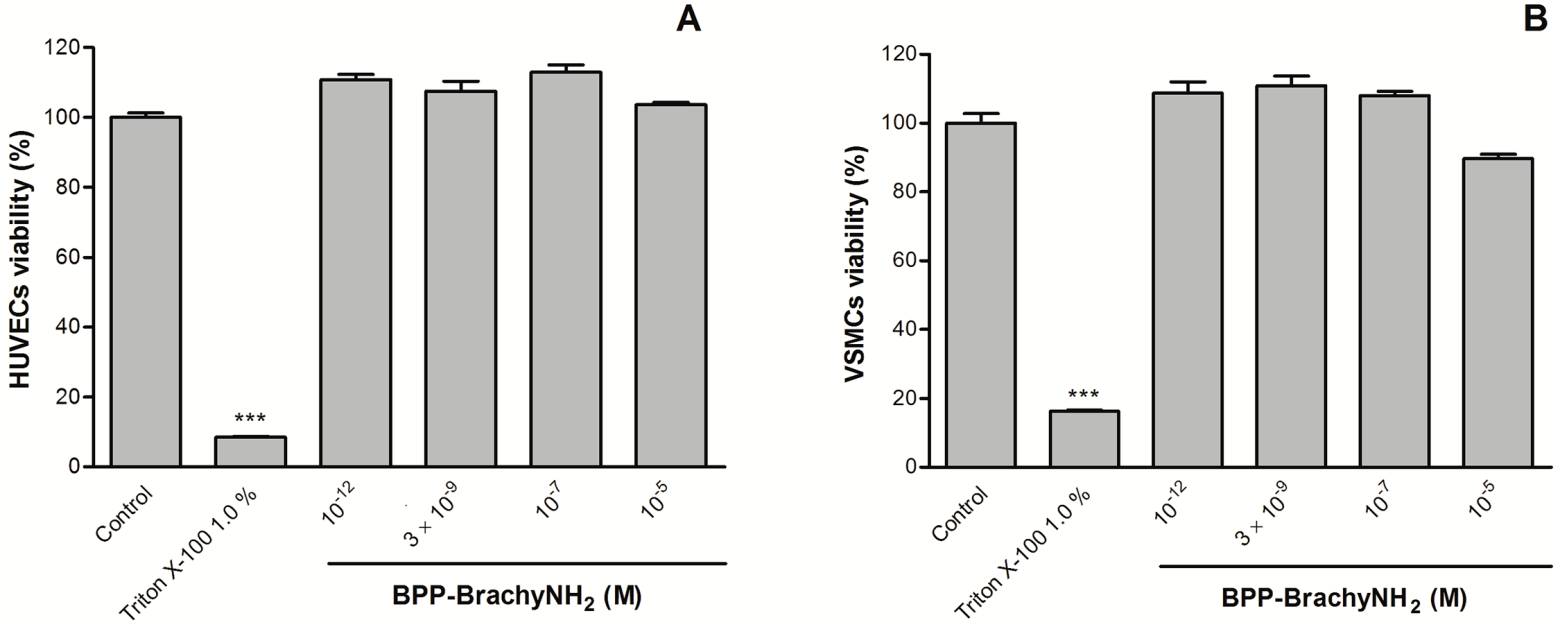
Effects of BPP-BrachyNH_2_ on cell viability of HUVECs and VSMCs cells. HUVECs (A) or VSMCs (B) were incubated with different concentrations of BPP-BrachyNH_2_ for 24 hours and then with MTT for 4 hours. Formazan crystals were dissolved in DMSO. The control group was treated with culture medium only. Data are mean ± SEM (n = 3). ^***^ *p* < 0.001 versus control.

The MTT assay was performed in order to evaluate the cell viability of HUVECs and aortic vascular smooth muscle cells in the presence of BPP-BrachyNH_2_. No cytotoxic effects were observed for the concentration range assayed (Fig 7), suggesting that vascular cell damage does not contribute to the vasodilatation and increases in NO induced by BPP-BrachyNH_2_.

## Discussion

The major finding of this study is that the peptide BPP-BrachyNH_2_ has a novel sequence and is the first PRO isolated from the skin secretion of the Brachycephalidae family, which opens for exploring amphibians as a source of new biomolecules. The BPP-BrachyNH_2_ is devoid of cytotoxicity and elicits endothelium-dependent vasodilatation mediated by NO. Interestingly, this study is the first peptidome characterized of a skin secretion in the Brachycephalidae family.

The presence of bradykinin (BK) and bradykinin-related peptides (BRPs) in the skin of amphibians has been related to the absence of the kallikrein-kinin system in these animals [36]. Therefore, the amphibian skin secretion of BK as well as BRPs potentiating the endogenous predator BK may lead to pronounced cardiovascular and gastrointestinal changes in the predator and function as a defense mechanism [37]. In the present study, the primary structures of WPPPKVSP (BPP-Brachy) and the amidated form thereof (BPP-BrachyNH_2_) were identified. Besides, there was no evidence for the presence of BK or any BRP in the skin secretion of *B*. *ephippium* (Fig 1B). However, the physiological importance of BPP-BrachyNH_2_ as a defense mechanism requires further investigation.

BK-potentiating activity was first described in hydroalcoholic extracts of *Bothrops jararaca* snake venom [8], and thereafter 25 BPPs have already been characterized [38]. The BPPs from *B*. *jararaca* commonly have a typical pyroglutamyl (Pyr) residue and proline-rich structure at the N- and C-terminus, respectively [39]. Nevertheless, the presence of the N-terminal pyroglutamyl residue has been demonstrated as a non-obligatory characteristic of BPP-similar peptides from different biological sources, but the presence of proline-rich residues mainly in the C-terminal region [17]. The BPP-BrachyNH_2_ lacks N-terminal pyroglutamic acid residues, and possesses two proline residues at the C-terminal portion (Fig 2). Table 1 shows that BPP-BrachyNH_2_ shares similarities with several other PROs from snakes, scorpions, spiders, and the frog *P*. *hypochondrialis*. The presence of tryptophan (W) followed by proline residues at N-terminal of the Lm-BPPs isolated from the scorpion *Lachesis muta*, is a common characteristic between these BPPs and BPP-BrachyNH_2_ [7]. Interestingly, the proline-tryptophan complexes possess very stable interactions and play important structural and interaction roles with other protein/peptide complexes. They are responsible for a wide variety of biological interactions and activation of cell signaling pathways [40].

**Table 1:** Proline-Rich Oligopeptides (PROs) from different biological sources.

Sequence	Name	Source	References
WPPPKVS**P**	BPP-Brachy	*Brachycephalus ephyppium*	This work
WPPRPQI**PP**	Lm-BPP 1	*Lachesis muta*	[7]
<EKWDPPPVS**PP**	Potentiator E	*Agkistrodon halys blomhoffii*	[53]
<EFRPSYQI**PP**	Phypo Xa	*Phyllomedusa hypochondrialis*	[37]
<EKWA**P**	*Bj*-BPP-5a	*Bothrops jararaca*	[9]
<EWPRPQI**PP**	*Bj*-BPP-9a	*B*. *jararaca*	[9]
<ESWPGPNI**PP**	*Bj*-BPP-10a	*B*. *jararaca*	[9]
<ENWPRPQI**PP**	*Bj*-BPP-10b	*B*. *jararaca*	[39,41]
<ENWPHPQI**PP**	*Bj*-BPP-10c	*B*. *jararaca*	[9]
<EWPRPTPQI**PP**	*Bj*-BPP-11a	*B*. *jararaca*	[41]
<EGRAPGPPI**PP**	*Bj*-BPP 11b	*B*. *jararaca*	[39,54]
<EARPPHPPI**PP**	*Bj*-BPP-11e	*B*. *jararaca*	[55]
<EWGRPPGPPI**PP**	*Bj*-BPP-12b	*B*. *jararaca*	[55]
<EGGWPRPGPEI**PP**	*Bj*-BPP-13a	*B*. *jararaca*	[39]
<EGGWPRPGPEI**PP**	BPP-III	*Bothrops neuwiedi*	[56]
<EARPPHPPI**PP**	BPP-XIe	*Bothrops jararacussu*	[57]
<ENWPHPQI**PP**	BPP-Xc	*B*. *jararacussu*	[57]
<EGGWPRPGPEI**PP**	BPP-XIIIa	*B*. *jararacussu*	[57]
<EARPPHPPIPPA**P**	BPP-AP	*B*. *jararacussu*	[57]
<EKWPPGKV**PP**	-	*Bothrops moojeni*	[38]
<ENWPRPGPEI**PP**	-	*B*. *moojeni*	[38]
<EKWPRPGPEI**PP**	BPP-BAX12	*B*. *moojeni; Bothrops atrox*	[38,58]
<ERWPHLEI**PP**	Cdt1b	*Crotalus d*. *terrificus*	[59]
<EAPWPDTIS**PP**	BPP-S	*Scaptocosa raptoria*	[44]
LRDYANRVINGGPVEAAG**PP**A	K12	*Buthus occitanus*	[42]

<E represents pyroglutamic acid; Bold represents typical C-terminal proline-rich sequences present in PROs.

Since the discovery of BPPs obtained from *B*. *jararaca* venom, they have been considered the first ACE inhibitors obtained from a natural source [8,9,41]. In animals other than snakes, inhibition of ACE activity has been found in venoms of the scorpions *Tityus serrulatus* [42] and *Buthus occitanus* [43], the spider *Scaptocosa raptoria* [44] and, more recently, in the skin secretion of *Phyllomedusa hypochondrialis*, the Brazilian tiger-legged monkey frog [37]. In this study, the ACE activity was determined by the fluorimetry measurement of His-Leu originated from hydrolysis of Hippuryl-His-Leu, a well recognized substrate of the C-domain of ACE [24]. BPP-BrachyNH_2_ induced a concentration-dependent decrease of ACE activity, and the results suggest that BPP-BrachyNH_2_ functionally acts as a BPP, as it was able to inhibit ACE activity (Fig 3). Therefore, the interactions between BPP-BrachyNH_2_ and both N- and C-domain of ACE were investigated by molecular docking. The evaluation by docking studies of peptide-enzyme was carried out based on *in vitro* results, which demonstrated a better competitive inhibition profile towards C-domain rather than N-domain. The relations between peptide and enzyme were extremely coordinated and guided *in silico* via amino acid residue side chains (Fig 4). Thus, the evidence from the *in silico* studies reinforces the ACE-inhibiting property of BPP-BrachyNH_2_ *in vitro*.

BPPs have been shown to cause vasodilatation in normotensive rats. The hypotensin TsHpt-I from the yellow scorpion *Tityus serrulatus* [45] and the *Bj*-BPP-5a from the *B*. *jararaca* venom induces both *in vitro* [16] and *in vivo* [15] vasodilatory effects. In this study, BPP-BrachyNH_2_ induced concentration-dependent relaxations in rat aortic rings, with E_max_ values around 2.0-fold higher than previously reported for *Bj*-BPP-5a and TsHpt-I. Despite captopril was a more potent inhibitor of ACE, the vasodilatation induced by captopril and BPP-BrachyNH_2_ was equipotent and of the same magnitude, suggesting that mechanisms other than ACE inhibition, appear to contribute to the relaxant effect of BPP-BrachyNH_2_ in rat aorta (Fig 5). These results were alike to previous reports on several targets for other BPPs-induced vasodilatory effects [19]. Moreover, despite a higher selectivity of captopril for ACE, when compared with BPPs, a direct correlation between BK potentiation, cardiovascular activity, and inhibition of the ACE has not been observed. Thus, this reinforces the possible involvement of distinct signaling pathways, which do not necessarily include inhibition of ACE [17].

The BPP-BrachyNH_2_-induced endothelium-dependent vasodilator effect suggests endothelium-derived mediators are involved in the vasodilatation. NO is an important endothelium-derived vasodilator and is pivotal in several biological processes [46–48]. The increase in NO production plays a pivotal role in the cardiovascular effects of BPPs. Thus, several *Bj*-BPPs were found to increase NO production either by activation of the AsS enzyme, resulting in conversion of L-citrulline to L-arginine, which increases the NO production in vivo [13,14], or the activation of G-protein coupled receptors (GPCRs) that triggers calcium-dependent mechanisms, which results in the increase of endothelial NO synthase (eNOS) activity [15,17,49]. Moreover, TsHpt-I from *T*. *serrulatus* venom, and *Bj*-BPP-5a from *B*. *jararaca* venom induced endothelium-dependent relaxations sensitive to eNOS inhibition in rat aorta [16,45]. In the present study, BPP-BrachyNH_2_ induced endothelium-dependent relaxations, which were mediated by NO as relaxation disappeared after inhibition of eNOS with L-NAME in rat aortic preparations (Fig 5).

Additional reports have demonstrated the *in vitro* increase of NO release in the presence of BPPs. *Bj*-BPP-5a was found by use of a chemiluminescence assay to increase NO release in HEK293 cells [15,17]. Other compounds, as hypotensins obtained from *T*. *serrulatus* venom increase NO release in murine cardiomyocytes, evaluated by confocal microscopy with the use of DAF-FM DA [50], a diaminofluorescein, which contains a 3-amino,4-aminomethyl,2-benzoic group linked to a fluorophore [51,52]. It is essentially non-fluorescent until it reacts with NO to form a highly fluorescent benzotriazole. The BPP-BrachyNH_2_ was able to increase fluorescence emission in DAF-FM DA-loaded aortic cross sections (Fig 6). These findings suggest that NO mediates the endothelium-dependent BPP-BrachyNH_2_-induced vasodilatation.

In conclusion, the peptide BPP-BrachyNH_2_ has a novel sequence and is the first BPP isolated from the skin secretion of the Brachycephalidae family. These findings open for exploring amphibians as a source of new biomolecules. The BPP-BrachyNH_2_ is devoid of cytotoxicity and elicits endothelium-dependent vasodilatation mediated by NO. This study not only reinforces the amphibians as an interesting source of important bioactive molecules, but it also emphasizes the worth of investigating their pharmacological, biotechnological and therapeutic potential for the treatment of endothelial dysfunction and cardiovascular diseases.
